# Rats Eat a Cafeteria-Style Diet to Excess but Eat Smaller Amounts and Less Frequently when Tested with Chow

**DOI:** 10.1371/journal.pone.0093506

**Published:** 2014-04-21

**Authors:** Timothy South, Nathan M. Holmes, Sarah I. Martire, R. Frederick Westbrook, Margaret J. Morris

**Affiliations:** 1 Department of Pharmacology, School of Medical Sciences, University of New South Wales, Sydney, Australia; 2 Department of Psychology, University of New South Wales, Sydney, Australia; Sapienza University of Rome, Italy

## Abstract

**Background:**

Obesity is associated with excessive consumption of palatable, energy dense foods. The present study used an animal model to examine feeding patterns during exposure to and withdrawal from these foods.

**Methods:**

Male Sprague Dawley rats were exposed to standard lab chow only (Chow rats) or a range of cafeteria-style foods eaten by people (Caf rats). After 1, 4, 7 and 10 weeks of diet in their home cage, rats were subjected to 24-hour test sessions in a Comprehensive Lab Animal Monitoring System (CLAMS). In the first two test sessions, Chow rats were exposed to standard lab chow only while Caf rats were exposed to a biscuit and high-fat chow diet. In the final two test sessions, half the rats in each group were switched to the opposing diet. In each session we recorded numbers of bouts, energy consumed per bout, and intervals between bouts across the entire 24 hours.

**Results:**

Relative to Chow rats, Caf rats initiated fewer bouts but consumed more energy per bout; however, their motivation to feed in the CLAMS declined over time, which was attributed to reduced variety of foods relative to their home cage diet. This decline in motivation was especially pronounced among Caf rats switched from the palatable CLAMS diet to standard lab chow only: the reduced energy intake in this group was due to a modest decline in bout frequency and a dramatic decline in bout size.

**Conclusions:**

Exposure to a cafeteria-diet, rich in variety, altered feeding patterns, reduced rats' motivation to consume palatable foods in the absence of variety, and further diminished motivation to feed when palatable foods were withdrawn and replaced with chow. Hence, variety is a key factor in driving excessive consumption of energy dense foods, and therefore, excessive weight gain.

## Introduction

The consumption of palatable energy dense foods has increased dramatically over the past forty years. This increased consumption has pushed rates of obesity to epidemic proportions among children, adolescents and adults across the world [1]. Excessive consumption of palatable energy dense foods leads to weight gain, but it is unclear how exposures to these foods influence eating patterns, particularly during the development of obesity. The fact that palatable energy dense foods are hedonically attractive, widely available and relatively cheap means that, compared to forty years ago, people today may eat more frequently (e.g., snacking), consume more each time they eat (e.g., increased portion size), or some combination of these factors. Alternatively, eating patterns may have changed very little in the last forty years: instead, people may have become overweight and obese in record numbers simply because the available foods are more energy dense.

Studies of eating patterns and how they change over time are difficult to conduct in people. It is difficult to ensure compliance, and therefore, to track eating across a prolonged period (like that which leads to obesity). Even when compliance is achieved, the nature of self-report data means it is difficult to verify the type and amount of foods that people eat. For these reasons, animal studies have been used to examine behavioural and biological factors that regulate feeding, and how exposure to palatable energy dense foods changes feeding patterns. In the latter respect, several investigators have reported that rodents, like people, prefer foods that are rich in fat and sugar, eat them to excess and become overweight [2]–[4]. We have shown that rats exposed to a range of the energy dense foods eaten by people also prefer these foods to chow, eat them to excess, become overweight, and display hallmarks of obesity [5]–[6]. Martire, Holmes, Westbrook and Morris (2013) recently examined how this cafeteria style diet affected feeding patterns [7]. Across several weeks of diet, rats exposed to the cafeteria diet initiated fewer meals (feeding immediately followed by grooming and sleeping, a sequence indicative of behavioural satiety) than rats exposed to standard lab chow. However during the early stages of diet exposure, rats exposed to the cafeteria diet snacked more than their chow-fed counterparts; and the early levels of snacking were directly related to terminal body weights.

Other evidence suggests that motivation to consume diets of varying palatability changes with extended exposure to a cafeteria-style diet. Naïve rats exposed to palatable foods for the first time consumed twice-as-much energy as rats with a history of exposure to these foods [8]. Conversely, when rats exposed to a cafeteria diet were subsequently switched to standard lab chow, they ate less than rats only fed chow, lost weight, and exhibited signs of hypothalamic-pituitary-adrenal (HPA) axis activation, evident as increased corticotrophin releasing factor (CRF) mRNA expression [8]–[9]. Whilst previous studies have examined the microstructure of feeding on palatable diets during the development of obesity, few have examined how feeding patterns change when a palatable diet is withdrawn. A notable exception is a study by Rogers (1985) who exposed rats to a diet composed of chow supplemented with white bread, milk chocolate and biscuits and then switched them to chow only [10]. He reported that the rats became hypophagic due to eating fewer meals and eating less on average at each meal. Rogers attributed this change in eating pattern, at least in part, to a negative contrast effect whereby the hedonic value of the chow was reduced because of the prior exposure to the highly palatable bread, chocolate and biscuits.

The present study had two aims. The first was to provide a further examination of feeding patterns among rats exposed to either a cafeteria diet or standard lab chow. Martire et al (2013) showed that exposure to a cafeteria-style diet influences the regularity of meals and snacks; however, the fact that feeding behaviour was examined in the home cage prohibited measurement of the energy density of each meal or snack. Here, we assessed feeding behaviour directed towards standard lab chow or palatable foods (biscuit and high-fat chow) using the Comprehensive Lab Animal Monitoring System (CLAMS), which permitted a more detailed examination of feeding frequency, amounts eaten, and the relationship between these measures. The second aim was to examine changes in feeding patterns among: (1) rats exposed to palatable foods for the first time; and (2) rats maintained on the cafeteria diet and then withdrawn from this diet for the first time (i.e., switched to standard lab chow only). Based on the results reported by South et al (2012), we expected that, relative to controls, rats exposed to palatable foods for the first time in the CLAMS would consume more, while rats withdrawn from the cafeteria diet for the first time in the CLAMS would consume less. Based on the results reported by Rogers (1985), we expected that rats withdrawn from the cafeteria diet would not only eat less but that this would be due to them eating fewer and smaller meals. Conversely, we expected that rats exposed to chow and given palatable foods would eat more and larger meals.

## Materials and Methods

### 1. Animal procedures

Male Sprague–Dawley rats (n = 48) were obtained from a commercial supplier (Animal Resource Centre, Perth, Australia) and maintained in a colony room kept at 23°C and on a 12 h light:12 h dark cycle (lights on at 07:00). Rats were housed in polypropylene cages (20 cm length ×32 cm width ×19 cm height) whose floors were covered with wood shavings. There were three rats per cage. Standard rat chow (CH, 11 kJ/g, energy 12% fat, 21% protein, 65% carbohydrate, Gordon's Specialty Stock feeds, NSW, Australia) and tap water were provided *ad libitum* throughout the experiment. Rats were handled daily and familiarized with the CLAMS in a single four hour session on one of these days. Following this acclimatization to the laboratory, half the rats (n = 24) were assigned to standard chow and half (n = 24) to the cafeteria diet which contained lab chow, modified chow [60% powdered chow mixed with 33% sweetened condensed milk and 7% saturated animal fat (pork lard)], as well as various foods (15.3 kJ/g, energy 32% fat, 14% protein, and 60% carbohydrate) selected from cakes, cookies, dim sims, pasta and meat pies. The foods selected were changed daily but always included one food (meat pies or dim sims) high in protein and two (cakes, biscuits, or pasta) high in carbohydrates and/or sugar. Food was provided daily at 17:00. Food intake was recorded once a week by measuring the amount of food provided at 17.00 hr and that remaining 24 hr later (with spillage taken into account).

#### Ethics Statement

All procedures were approved by the Animal Care and Ethics Committee of the University of New South Wales and were consistent with guidelines for animal research by the Australian National Health and Medical Research Council.

### 2. Feeding in the Comprehensive Lab Animal Monitoring System (CLAMS)

All rats were removed from their home cages and placed in individual plexiglass cages (60 cm length ×20 cm width ×20 cm height) of the Comprehensive Lab Animal Monitoring System (CLAMS; Columbus Instruments, Columbus, OH). During CLAMS monitoring, feeding was assessed using ground food placed in 24 metal floor feeders, each attached to the CLAMS plexiglass cages (two feeders/cage). Feeders rested on balances directly linked to a computer for measuring continuous food intake (every thirty seconds). During each of the CLAMS sessions, cumulative food intake was automatically recorded for 24 h. A bout was defined as a period of continuous eating: the minimum bout duration was the resolution at which data were collected in the CLAMS, 30 s; two consecutive 30 s periods in which feeding occurred was defined as a single 60 s bout; three consecutive 30 s periods in which feeding occurred was defined as a single 90 s bout; and so forth.

All rats were tested in the CLAMS apparatus across four sessions. These sessions occurred one, four, seven and 10 weeks after diet exposure, each lasted 24 hours, and commenced at 15:00. In the first two sessions, rats were exposed to foods consistent with their home-cage history. Rats maintained on the cafeteria diet in their home cage, termed ‘Caf’ rats, were exposed to two palatable (PAL) foods: powdered, sweetened high fat chow (14% Fat, 40% Carbohydrate, 8% Protein) and powdered shortbread cookies (20% Fat, 71% Carbohydrate, 6.5% Protein). Rats maintained on standard lab chow in their home cage, termed ‘Chow’ rats, were exposed to the same standard lab chow in the CLAMS but in powdered form (4% Fat, 50% Carbohydrate, 14% Protein). In the final two sessions, half of the Caf rats were again exposed to biscuit and high-fat chow (Group Caf-PAL); and half of the Chow rats were again exposed to standard lab chow (Group Chow-CHOW). Remaining Caf rats were exposed to standard lab chow in the CLAMS for the first time (Group Caf-CHOW) and remaining Chow rats were exposed to palatable biscuit and high-fat chow for the first time (Group Chow-PAL; see Table 1). Water was continuously available in the clams.

**Table 1:** Description of groups used in the current experiment.

	Palatable diet in CLAMS	Chow only in CLAMS
Cafeteria diet in home cage	Caf-PAL	Caf-CHOW
Chow only in home cage	Chow-PAL	Chow-CHOW

### 3. Statistical Analysis

#### 3a. Home cage 24-hour energy intake (kJ) and body weight

During each of the weeks in which rats were subjected to CLAMS test sessions, one 24-hour period was selected for measurement of home cage energy intake. This consisted in weighing the food prior to placement in the home cage, and after a period of 24 hours, any food leftover was weighed. The difference in weight of each food was used to calculate total 24-hour energy intake for the three rats in each home cage. The average intake per cage was analyzed by ANOVA with a between-subjects factor of group (Caf versus Chow) and a within-subjects factor of time (across four time points of the study). The body weights of individual rats were also recorded during these same periods and analyzed by ANOVA with a between-subjects factor of group (Caf versus Chow) and a within-subjects factor of time (across four time points of the study).

#### 3b. Feeding in Caf and Chow rats

We conducted three analyses. Analysis 1 examined feeding patterns in Caf and Chow rats, focusing on feeding in Groups Caf-PAL and Chow-CHOW across the four CLAMS test sessions, and within each CLAMS test session. Analysis 2 examined the effect of a diet switch on feeding by Caf and Chow rats. Here, we focused on changes in feeding patterns in Group Chow-PAL relative to Group Caf-PAL, and Group Caf-CHOW relative to Group Chow-CHOW. Analysis 3 examined the more acute effects of a diet switch. We directly compared feeding in Group Chow-PAL during its first exposure to the palatable biscuit and high-fat chow in week 7 with feeding in Group Caf-PAL during its first exposure to the palatable biscuit and high-fat chow in week 1. We also compared feeding in Group Caf-CHOW during its first exposure to standard lab chow in the CLAMS in week 7 with feeding in Group Chow-CHOW during its first exposure to chow in the CLAMS in week 1.

Analyses 1 and 2 progressed in four stages. First, levels of energy intake (kJ) were submitted to ANOVA with a between-subjects factor of group (Caf-PAL vs. Chow-CHOW) and within-subjects factors of test session (Sessions 1–4) and time (blocks of four hours within a session). Second, numbers of bouts and energy intake per bout were submitted to ANOVA with group as a between-subjects factor (Caf-PAL vs. Chow-CHOW) and test session as a within-subjects factor (Sessions 1–4). Third, distributions of inter-bout intervals and bout sizes were generated by pooling these data across rats within groups, and the Kolmogorov-Smirnov (abbreviated to K-S) test was used to assess differences in the distributions between the groups. Finally, average bout size was plotted against average inter-bout interval for individual rats in each group, and Pearson product-moment correlations were used to assess the degree of linearity in the relationship between these variables.

In Analysis 3, single factor ANOVAs were used to separately compare Groups Caf-PAL and Chow-PAL, and Groups Caf-CHOW and Chow-CHOW on each of the following measures: total energy intake, numbers of bouts, and energy intake per bout (bout size). Thereafter, average bout size was plotted against average inter-bout interval for individual rats in each group. The degree of linearity in the relationship between these variables was assessed using Pearson product-moment correlations.

At each stage of each analysis, the criterion for rejection of the null hypothesis was set at alpha = .05.

## Results

### 1. Home cage 24-hour energy intake (kJ) and body weight

**Figure 1:**
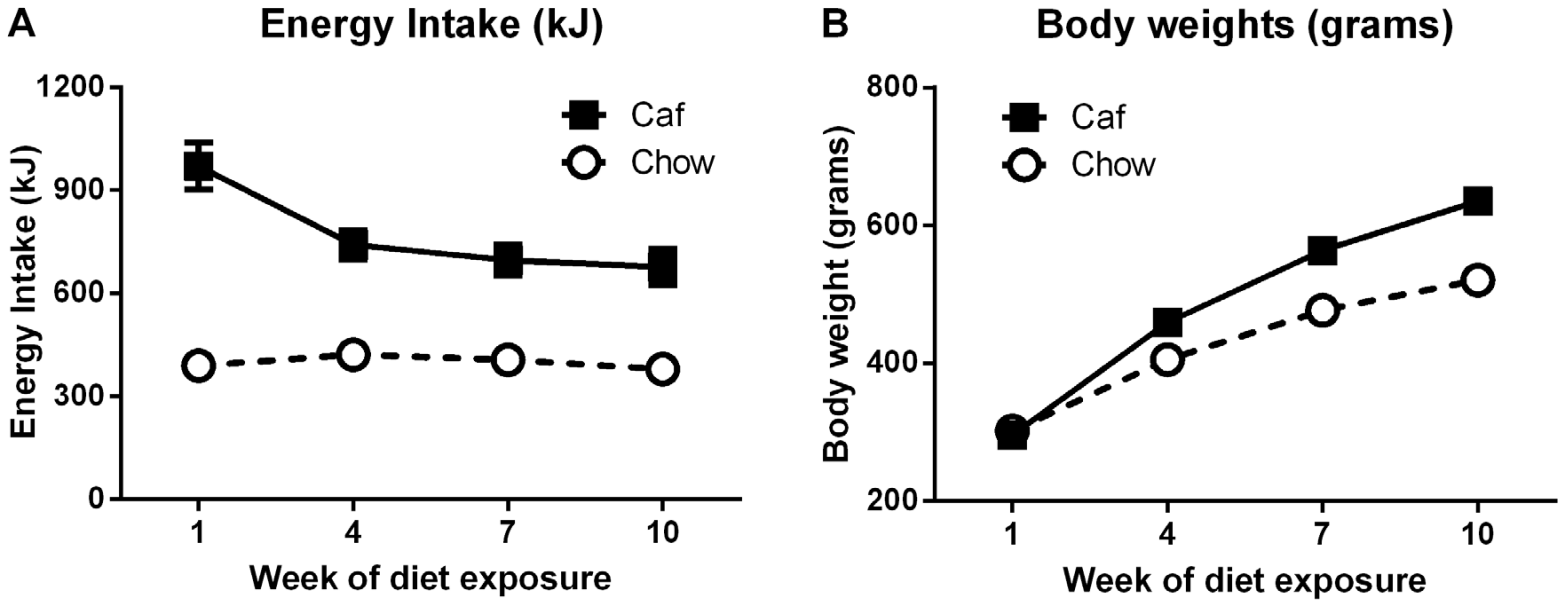
Home cage energy intake. Average home cage 24-hour energy intake (kJ, **A**) and individual body weights of Caf and Chow rats (**B**) across the duration of the experiment. Each data point represents the mean (+SEM) for the particular unit of analysis.

Figure 1 shows average energy intake (kJ, 1A) and body weight (1B) for all Caf and all Chow rats across the duration of the experiment. Analysis of the energy intake data confirmed that, overall, Caf rats consumed more energy than Chow rats, F(1,6) = 228.59, p<.01. Critically, the size of the difference in energy intake between the two groups was most pronounced during the early weeks of diet exposure, evidenced by a statistically significant group x (linear) interaction, F(1,6) = 6.86, p<.05. However, even in the final week of diet exposure, it is clear that, Caf rats consumed almost double the amount of energy as Chow rats. Analysis of body weight data also showed that, averaged over groups, there was a linear increase in body weight across weeks of the study, *F*(1,46) = 2059.77, p<.01, and that the rate of increase was greater for Caf than Chow rats, group x (linear) interaction, *F*(1,46) = 93.12, p<.01. Consequently, Caf rats became significantly heavier than Chow rats, *F*(1,46) = 49.84, p<.01. In sum, Caf rats consumed more energy and gained weight more rapidly than Chow rats.

### 2. Feeding in Caf and Chow rats

#### 2a. Changes in energy intake (kJ) between and within 24-hour CLAMS test sessions

**Figure 2:**
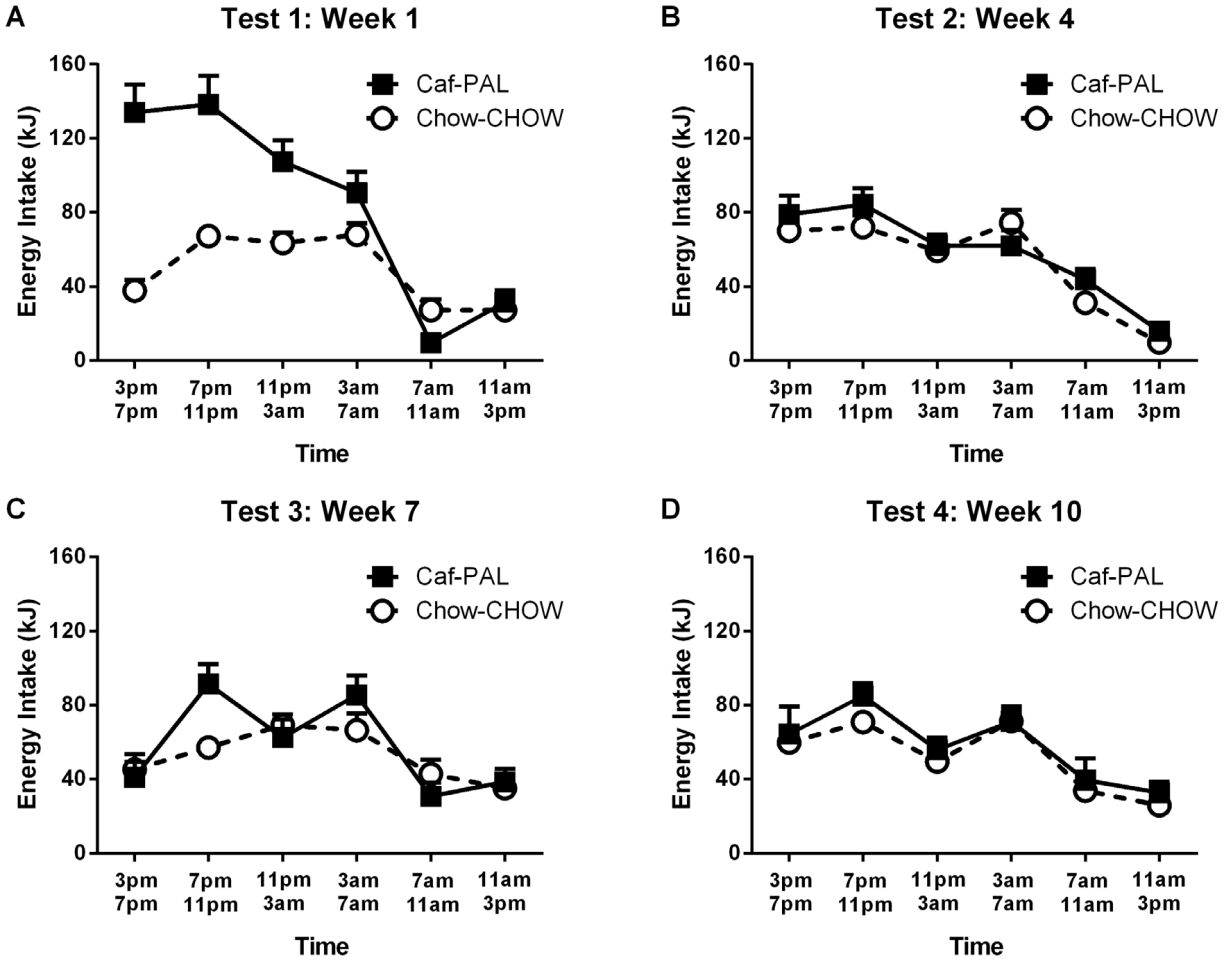
CLAMS energy intake. (**A–D**) Energy intake (kJ) for Caf-PAL and Chow-CHOW rats in each of the 24-hour CLAMS test sessions. Each data point is the mean (+SEM) energy intake in a block of four hours.

Figure 2 shows energy intake (kJ) across blocks of four hours for Caf-PAL and Chow-CHOW rats in each of the CLAMS test sessions. It is clear that Caf rats consumed more kJ than Chow rats when they were first introduced to the CLAMS in week 1 (2A), but that energy intake in the two groups was equivalent thereafter as energy intake declined across and within test sessions (2B–D). The statistical analysis confirmed a significant decline in energy intake between-, *F*(1,22) = 37.26, p<.01, and within-sessions, *F*(1,22) = 81.27, p<.01. Critically, both of these effects interacted with Group, smaller *F*(1,22) = 8.22, p<.01; and the three-way interaction between Group, test and time was also significant, *F*(1,22) = 26.94, p<.01.

To determine the source of the three-way interaction, the data from each test session were submitted to ANOVA with factors of Group and time. In Week 1, Caf rats consumed more energy than Chow rats, *F*(1,22) = 126.05, p<.01. A significant group x time interaction confirmed that this difference between the two groups was most pronounced during the early stages of the test session, *F*(1,22) = 33.01, p<.01. Analyses of the data from each of the remaining test sessions (in weeks 4, 7 and 10) showed that energy intake declined over time, smallest *F*(1,22) = 12.54, p<.01. The rate of this decline was equivalent for Caf and Chow rats: the Group x time interaction was not significant, *Fs*<1; and overall, there was no significant difference in energy intake between the two groups, *Fs*<2.36.

#### 2b. Changes in bout number and bout size between sessions

**Figure 3:**
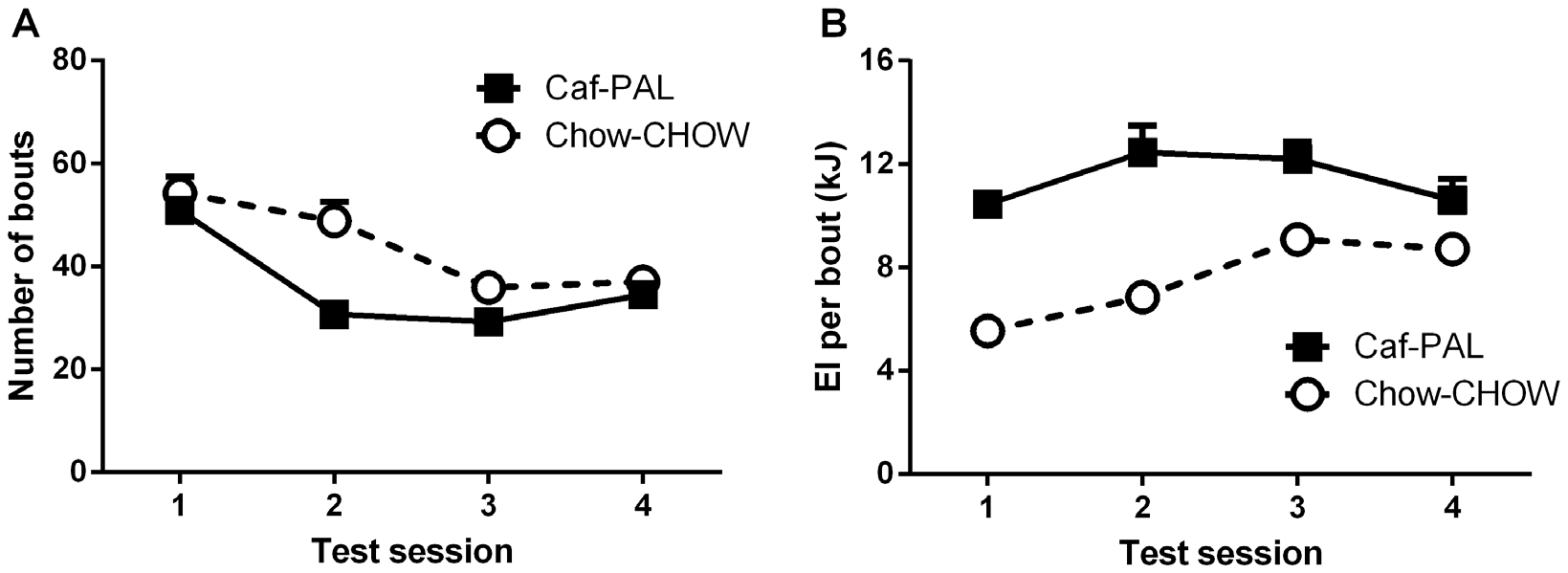
Bout frequency and size. Total numbers of bouts (**A**) and average bout size (**B**) in each of the CLAMS test sessions for Caf-PAL and Chow-CHOW rats. Each data point represents the mean (+SEM) for the entire session.

*Number of bouts* Figure 3A shows the total numbers of bouts in each of the CLAMS test sessions for Caf-PAL and Chow-CHOW rats. It is clear that the numbers of bouts declined across test sessions, and that this decline was more pronounced for Caf-PAL than Chow-CHOW rats. The analysis of these data revealed significant linear and quadratic components to the decline in bout numbers across test sessions, smaller *F*(1,22) = 20.02, p<.01, the latter of which interacted with Group, *F*(1,22) = 7.06, p<.05. This reflects the finding that Caf-PAL rats had fewer bouts than Chow-CHOW rats in the middle test sessions; indeed, Caf-PAL rats had fewer bouts than Chow-CHOW rats overall, *F* (1,22) = 7.32, p<.05.

*Bout size* Figure 3B shows the average bout size in each of the CLAMS test sessions for Caf-PAL and Chow-CHOW rats. Bout size increased across test sessions, *F* (1,22) = 11.73, p<.01, and the rate of this increase was greater for Chow-CHOW than Caf-PAL rats, *F* (1,22) = 10.76, p<.01. However, overall, Caf-PAL rats had larger bouts than Chow-CHOW rats, *F* (1,22) = 52.55, p<.01.

In summary, Caf-PAL rats consumed more energy than Chow-CHOW rats in the CLAMS during the early but not late stages of diet exposure. Despite equivalent energy intake during the late stages of diet exposure, the two groups differed in the way energy was obtained: Caf-PAL rats initiated fewer bouts than Chow-CHOW rats, but on average, each bout was higher in energy.

#### 2c. Distributions of waiting times (between bouts) and bout sizes

**Figure 4:**
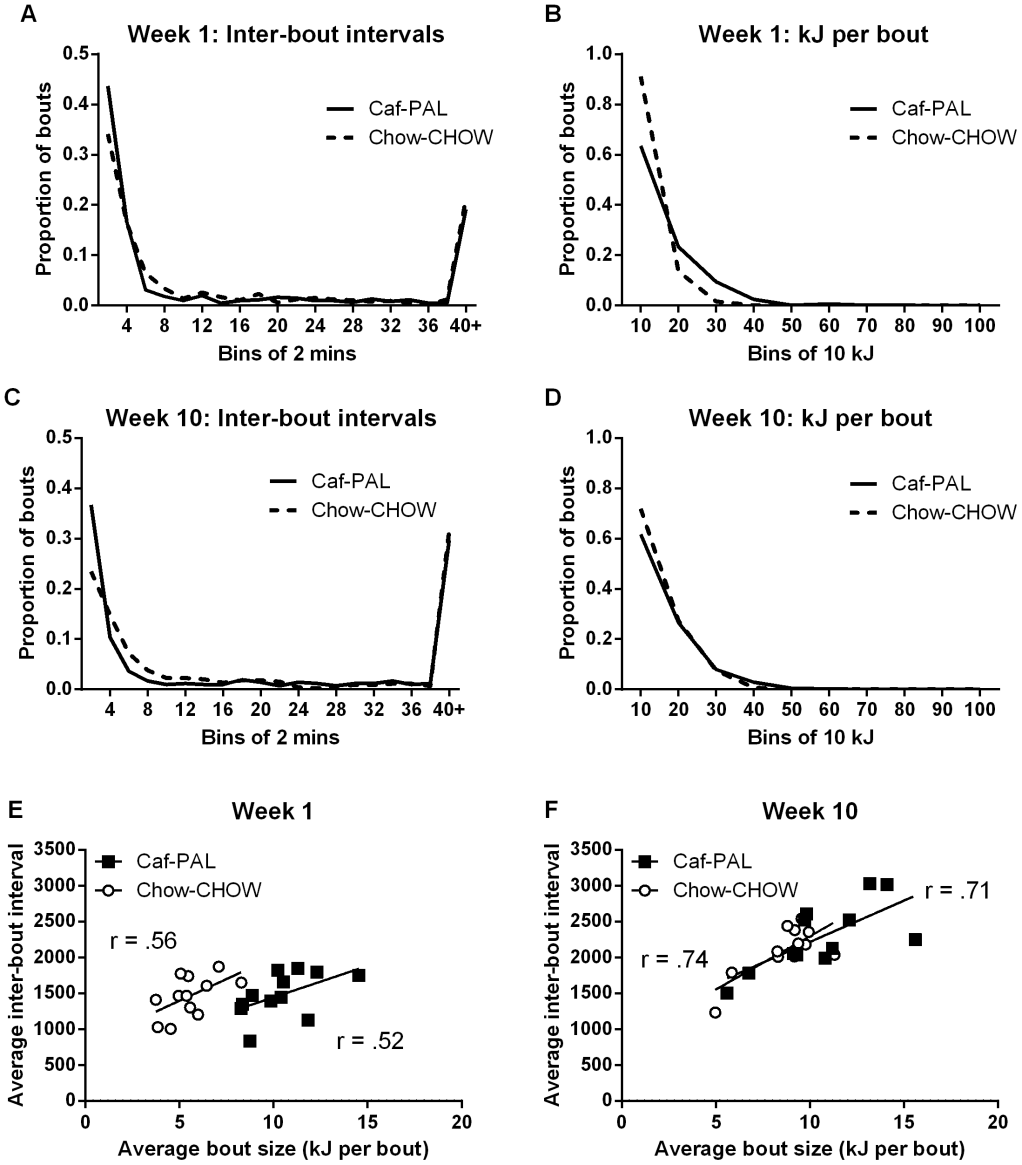
Distributions of inter-bout intervals and bout sizes. (**A & B**) Distributions of inter-bout intervals (A) and bout sizes (B) for Caf-PAL and Chow-CHOW rats during the first CLAMS test session (week 1). (**C & D**) Distributions of inter-bout intervals (C) and bout sizes (D) for Caf-PAL and Chow-CHOW rats during the last CLAMS test session (week 10). (**E & F**) Average bout size (kJ) versus average inter-bout interval for individual Caf-PAL and Chow-CHOW rats in the first (week 1, E) and final (week 10, F) CLAMS test sessions.

Figures 4A–D show the distributions of inter-bout intervals and bout sizes for Caf-PAL and Chow-CHOW rats during the first and last test sessions. In the first test session, Caf-PAL rats showed a leftward shift in the distribution of inter-bout intervals (Figure 4A), K-S test <.001, and a rightward shift in the distribution of bout sizes (Figure 4B), K-S test <.001. These shifts reflect the fact that Caf-PAL rats had proportionally more short inter-bout intervals (<2 min), and proportionally more bouts of larger size.

In the final test session, Caf-PAL rats again showed a leftward shift in the distribution of inter-bout intervals (Figure 4C), K-S test <.001, but there was no difference in the distribution of bout sizes between the two groups (Figure 4D), K-S test >.05. Inspection of the figure shows that the latter result was due to a rightward shift in the distribution for Chow-CHOW rats from week 1 to week 10: that is, an increase in bout size among Chow-CHOW rats as opposed to a decrease in bout size among Caf-PAL rats.

In summary, exposure to a cafeteria diet in the home cage led to a persistent increase in the number of short inter-bout intervals (<2 min) relative to chow-fed controls, and a transient increase in the numbers of larger bouts (>10 kJ).

#### 2d. Relationship between energy intake and wait times

Figures 4E and F show average bout size plotted against average inter-bout interval for individual Caf-PAL and Chow-CHOW rats in the first (4E) and final (4F) test sessions. Both groups showed a positive relationship between these variables in each test session: this relationship approached significance in the first test session, *r* = .52 for Caf-PAL rats and *r* = .56 for Chow-CHOW rats, larger *t*(10) = 2.12, p = .06, and was highly significant in the final test session, *r* = .74 for Caf-PAL rats and *r* = .71 for Chow-CHOW rats, smaller *t*(10) = 3.21, p<.01. Thus, rats that consumed more energy per bout waited longer before initiating the next bout or, alternatively, rats that waited longer between bouts consumed more energy per bout.

### 3. The effect of a diet switch on feeding in Caf and Chow rats

#### 3a. Changes in energy intake (kJ) between and within 24-hour CLAMS test sessions

**Figure 5:**
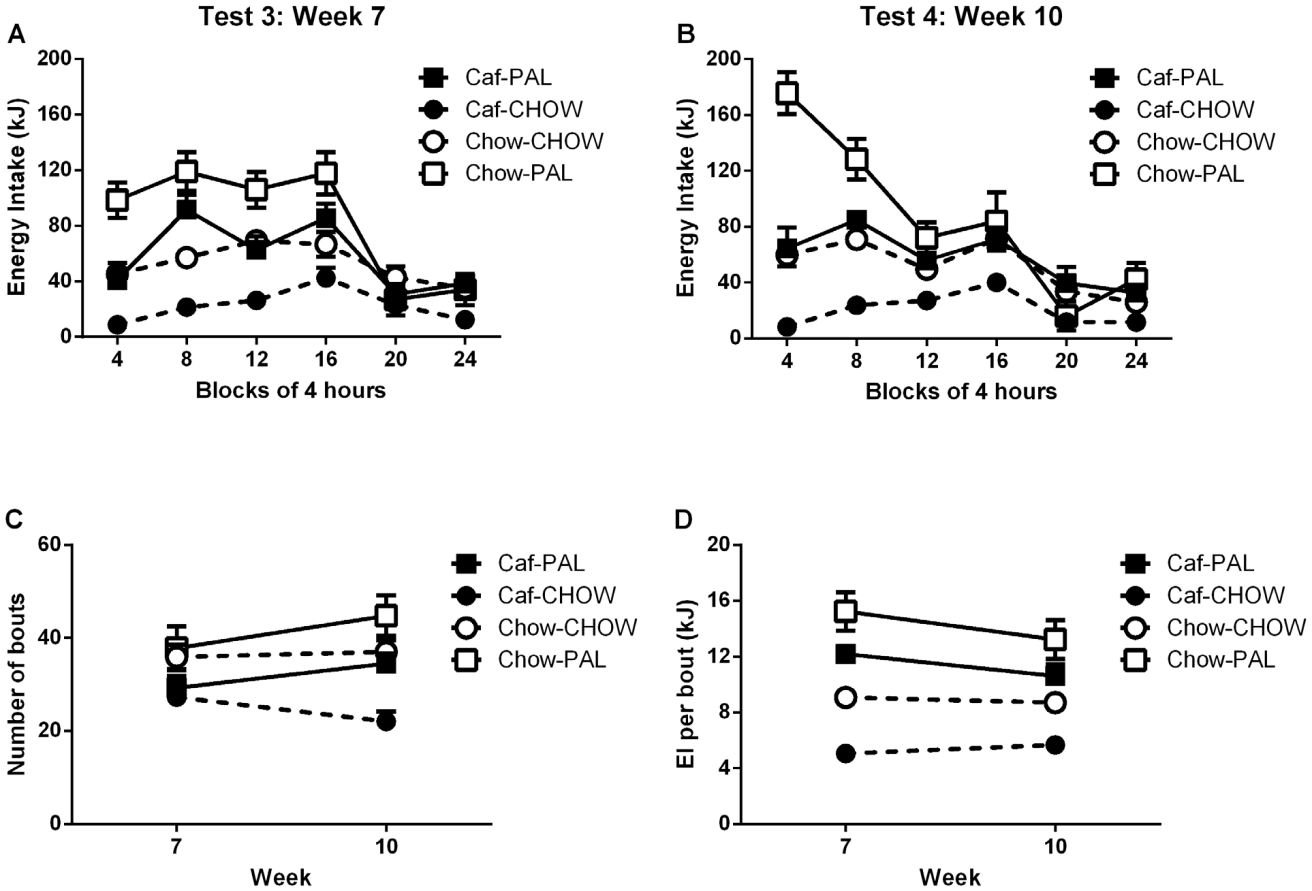
Post-switch CLAMS energy intake. (**A**) Energy intake (kJ) for each of the four groups during the 24-hour CLAMS test sessions in week 7. (**B**) Energy intake (kJ) for each of the four groups during the 24-hour CLAMS test sessions in week 10. Each data point in A and B is the mean (+SEM) energy intake in a block of four hours. (**C**) Total numbers of bouts for each of the four groups during test sessions in weeks 7 and 10. (**D**) Average bout size for each of the four groups during test sessions in weeks 7 and 10. Each data point in C and D represents the mean (+SEM) for an entire session.

Figures 5A and B show energy intake (kJ) across blocks of four hours for each of the four groups during the post-switch test sessions. As noted above, at each of these two time points, Caf-PAL and Chow-CHOW rats were equivalent with respect to both total energy intake, and the pattern of energy intake across 24 hours of testing. Relative to each of these groups, there were clear differences in feeding behaviour among Chow rats tested with palatable foods (Chow-PAL) and Caf rats tested with standard lab chow (Caf-CHOW): Chow-PAL rats consumed the most energy, Caf-CHOW rats consumed the least energy, and these effects were most apparent during the early stages of each test session. Statistical analysis revealed a significant main effect of Caf-vs-Chow, *F*(1,44) = 109.22, p<.01, and a two-way Caf-vs-Chow x switch interaction, *F*(1,44) = 158.83, p<.01.

Higher-order interactions involving both of these factors confirmed that the elevated energy intake in Group Chow-PAL and reduced energy intake in Group Caf-CHOW were most pronounced during the early stages of each test session, evidenced by significant three-way Caf-vs-Chow x switch x time interaction, *F*(1,44) = 79.92, p<.01); and that the size of these differences increased from the first to the second test session, evidenced by a significant four-way Caf vs. Chow x switch x time x test interaction, *F*(1,44) = 4.60, p = .05. Post-hoc comparisons confirmed that in the first block of four hours in each test session, Chow-PAL rats consumed more energy than Caf-PAL rats, smaller *F*(1,22) = 14.29, while Caf-CHOW rats consumed less energy than Chow-CHOW rats, smaller *F*(1,22) = 16.24.

In summary, when exposed to palatable foods, Chow rats consumed more energy than Caf rats; and when exposed to chow, Caf rats consumed less energy than Chow rats.

#### 3b. Changes in bout number and bout size between sessions

*Number of bouts* Figure 5C shows the total numbers of bouts for each of the four groups during testing. It is clear that, independently of the foods available at test, Caf rats had fewer bouts than Chow rats, *F*(1,44) = 16.98, p<.01. The two-way Caf vs. Chow x switch interaction was significant, *F*(1,44) = 5.47, p<.05, as was the three-way Caf vs. Chow x switch x test interaction, *F*(1,44) = 6.35, p<.05. These interactions imply that the overall difference in bout numbers between Caf and Chow rats was largely due to low numbers of bouts in Caf rats switched from palatable foods to standard lab chow (Caf-CHOW), especially in the final test session. Post-hoc tests of bout numbers in the final session revealed that Caf-CHOW rats had fewer bouts than Chow-CHOW rats, *F*(1,22) = 20.36, but Caf-PAL and Chow-PAL rats did not significantly differ, *F*(1,22) = 4.43.

*Bout size* Figure 5D shows the average bout size for each of the four groups during testing. It is clear that in both test sessions, Chow-PAL rats had the largest (most energy dense) bouts, Caf-PAL and Chow-CHOW rats had moderately sized bouts, and that Caf-CHOW rats had the smallest bouts. ANOVA revealed a main effect of Caf vs. Lean, *F*(1,44) = 22.71, p<.01, which was moderated by a two-way Caf vs. Chow x switch interaction, *F*(1,44) = 72.68, p<.01. Post-hoc tests confirmed that average bout size was smaller for Caf-CHOW rats compared to Chow-CHOW rats, *F*(1,22) = 39.15, but Caf-PAL and Chow-PAL rats did not significantly differ, *F*(1,22) = 5.46.

In summary: Caf rats initiated fewer bouts, consumed less energy per bout, and therefore, less energy overall than Chow rats. These differences were largely due to a persistent reduction in consumption of lab chow among Caf rats used to eating a range of palatable foods; and partly due to enhanced consumption of the palatable foods among Chow rats used to eating lab chow.

#### 3c. Distributions of waiting times (between bouts) and bout sizes

**Figure 6:**
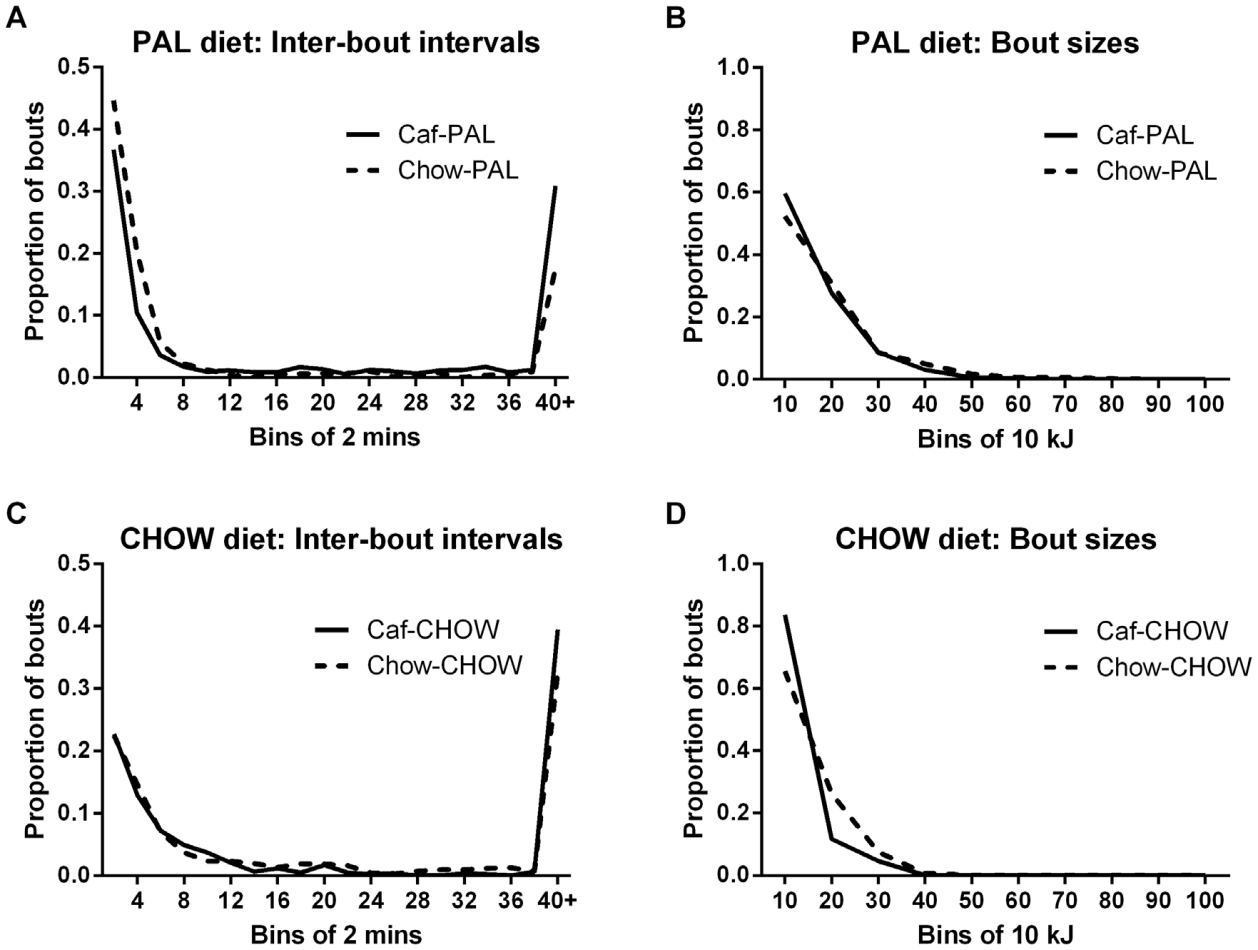
Post-switch distributions of inter-bout intervals and bout sizes. Distributions of inter-bout intervals (left, A & C) and bout sizes (right, B & D) for groups tested with either palatable foods (top, A & B) or standard lab chow (bottom, C & D). The data shown are from the final test session only (in week 10).

Figure 6 shows the distributions of inter-bout intervals (left) and bout sizes (right) for groups tested with either palatable foods (top) or standard lab chow (bottom). The data shown are from the final test session only. Among rats tested with palatable foods, there was a leftward shift in the distribution of inter-bout intervals for Chow-PAL rats relative to Caf-PAL rats (Figure 6A), K-S test <.001; and a small but statistically significant rightward shift in the distribution of bout sizes (Figure 6B), K-S test <.01. In contrast, among rats tested with standard lab chow, there was a leftward shift in the distribution of bout sizes for Caf-CHOW rats relative to Chow-CHOW rats (Figure 6C), K-S test <.001; but there was no difference between these two groups in the distribution of inter-bout intervals (Figure 6D), K-S test >.05.

Thus, compared to Caf rats with a history of exposure to palatable foods, Chow rats tested with palatable foods consumed similar sized bouts, but had proportionally more wait times of shorter duration (from one bout to the next); and compared to Chow rats with a history of exposure to chow only, Caf rats tested with chow showed a similar distribution of waiting times to the next bout, but had proportionally more bouts of smaller size.

#### 3d. Consumption of biscuit and high fat chow by rats offered palatable foods

**Figure 7:**
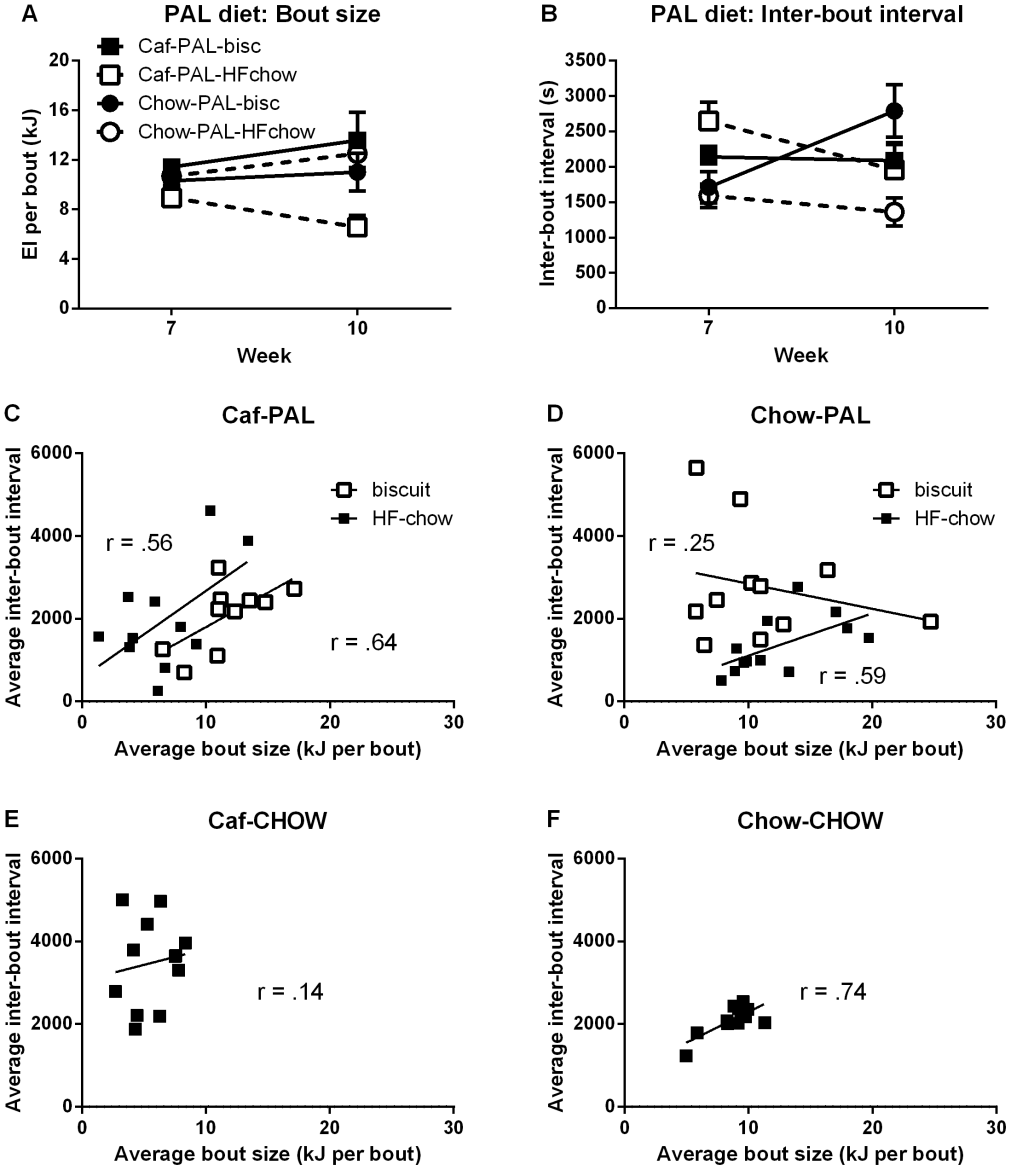
Palatable food consumption in the CLAMS. (**A**) Average bout size (kJ) for Caf-PAL and Chow-PAL rats, separated for bouts consisting of either biscuit only or high-fat chow only. (**B**) Average wait time to the next bout for Caf-PAL and Chow-PAL rats, separated for bouts consisting of either biscuit only or high-fat chow only. Each data point in A and B represents the mean (+SEM) for an entire session. (**C & D**) Average bout size versus average inter-bout interval for individual Caf-PAL (C) and Chow-PAL rats (D) in the final test session, again separated according to bout identity. (**E & F**) Average bout size versus average inter-bout interval for individual Caf-CHOW (E) and Chow-CHOW rats (F) in the final test session.

Figure 7 shows the average size (kJ, 7A) and average wait time (7B) to the next bout for Caf-PAL and Chow-PAL rats, separated for bouts consisting of either biscuit only or high-fat chow only. Caf-PAL rats tended to have larger biscuit bouts relative to high-fat chow, especially in the final test session; whereas biscuit and high-fat chow bouts for Chow-PAL rats were similar in terms of their average size. In contrast, among Caf-PAL rats, the average waiting time to the next bout was the same following a bout of biscuit or a bout of high-fat chow; but among Chow-PAL rats, the average waiting time to the next bout was longer following a bout consisting of biscuit versus a bout consisting of high-fat chow. These impressions were supported statistically.

*Percent of total energy intake from biscuit* The percentage of total energy intake obtained through consumption of biscuit differed between Caf-PAL and Chow-PAL rats: Averaged across the final two test sessions, these percentages were 72% and 49% for Caf-PAL and Chow-PAL rats, respectively. A two-way ANOVA of these data revealed a main effect of group, *F*(1,22) = 6.44, p<.05, but no effect of test session and no group x test interaction, *Fs*<1.

*Average bout size* Averaged across all rats in the two groups, the average size of biscuit bouts was greater than that of high-fat chow, *F*(1,22) = 5.91, p<.05. The size of biscuit bouts relative to high-fat chow bouts differed between the two groups, evidenced by a significant two-way food x group interaction, *F*(1,22) = 12.29, p<.01; and this difference was most evident in the final test session, evidenced by a significant three-way food x group x test interaction, *F*(1,22) = 6.24, p<.05. To identify the source of these interactions, the data from each test session were submitted to ANOVA with factors of group and food. In the first post-switch test session (week 7), there was no overall difference in bout size for biscuit versus high-fat chow, *F*(1,22) = 2.4, no overall difference in bout size between the two groups, *F*<1, and no group x food interaction, *F*(1,22) = 4.38, Fc = 5.76. In the final test session, the main effects of biscuit versus high-fat chow bout, *F*(1,22) = 5.34, and group, *F*<1, were again not significant; but there was a significant interaction between these factors, *F*(1,22) = 11.59, p<.05, consistent with larger biscuit bouts than high-fat chow bouts among Caf-PAL rats but not Chow-PAL rats.

*Inter-bout intervals* Overall, there was no difference in average wait time to the next bout following a bout consisting of biscuit or a bout consisting of high-fat chow, *F*(1,22) = 1.44. However, there was a significant test x food interaction, *F*(1,22) = 10.75, p<.01, suggesting that differences in waiting times following a biscuit bout or a high-fat chow bouts changed across testing. Inspection of Figure 7B suggests that this change was due to a decrease in post-high-fat-chow wait times among Caf-PAL rats and an increase in post-biscuit wait times among Chow-PAL rats. However, the two-way food x group interaction only approached significance, *F*(1,22) = 3.86, and the three-way food x group x test interaction was not significant, *F*(1,22) = 1.27.

Figures 7C and D show average bout size plotted against average inter-bout interval for individual Caf-PAL (7C) and Chow-PAL rats (7D) in the final test session, again separated according to bout identity (biscuit or high-fat chow). Among Caf-PAL rats (7C), there was a positive relationship between the amount of biscuit consumed and post-biscuit wait times, *t*(10) = 2.62, p<.025; the relationship between amount of high-fat-chow consumed and post-high-fat-chow wait times approached significance, *t*(10) = 2.13, p = .06. Chow-PAL rats (7D) also showed a clear positive relationship between amount of high-fat-chow consumed and post-high-fat-chow wait times, *t*(10) = 2.30, p<.025; but these rats showed no relationship between the amount of biscuit consumed and post-biscuit wait times, *t*<1.

Figures 7E and F show the corresponding plots for individual Caf-CHOW and Chow-CHOW rats, respectively. Here-Chow-CHOW rats showed a positive relationship between amount of chow consumed and wait times between chow bouts, *t*(10) = 3.58, p<.01, whereas Caf-CHOW rats showed no such relationship, *t*<1.

In summary: Caf-PAL rats consumed more biscuit than high-fat chow, both overall and in terms of average bout size, at least by the final session of testing; however, the average waiting times following consumption of either food were equivalent. Chow-PAL rats showed the opposite pattern: they ate equal amounts of biscuit and chow, both overall and in terms of average bout size, but waited longer to the next bout after having eaten biscuit. With the exception of Chow-PAL and Caf-CHOW rats, all groups showed a positive relationship between bout size and wait times: rats that had larger average bout sizes had longer average wait times to the next bout. This relationship was not evident with respect to consumption of biscuit by Chow-PAL rats, or consumption of chow by Caf-CHOW rats.

### 4. Feeding in switch groups in week 7 relative to Caf and Chow rats in week 1

**Figure 8:**
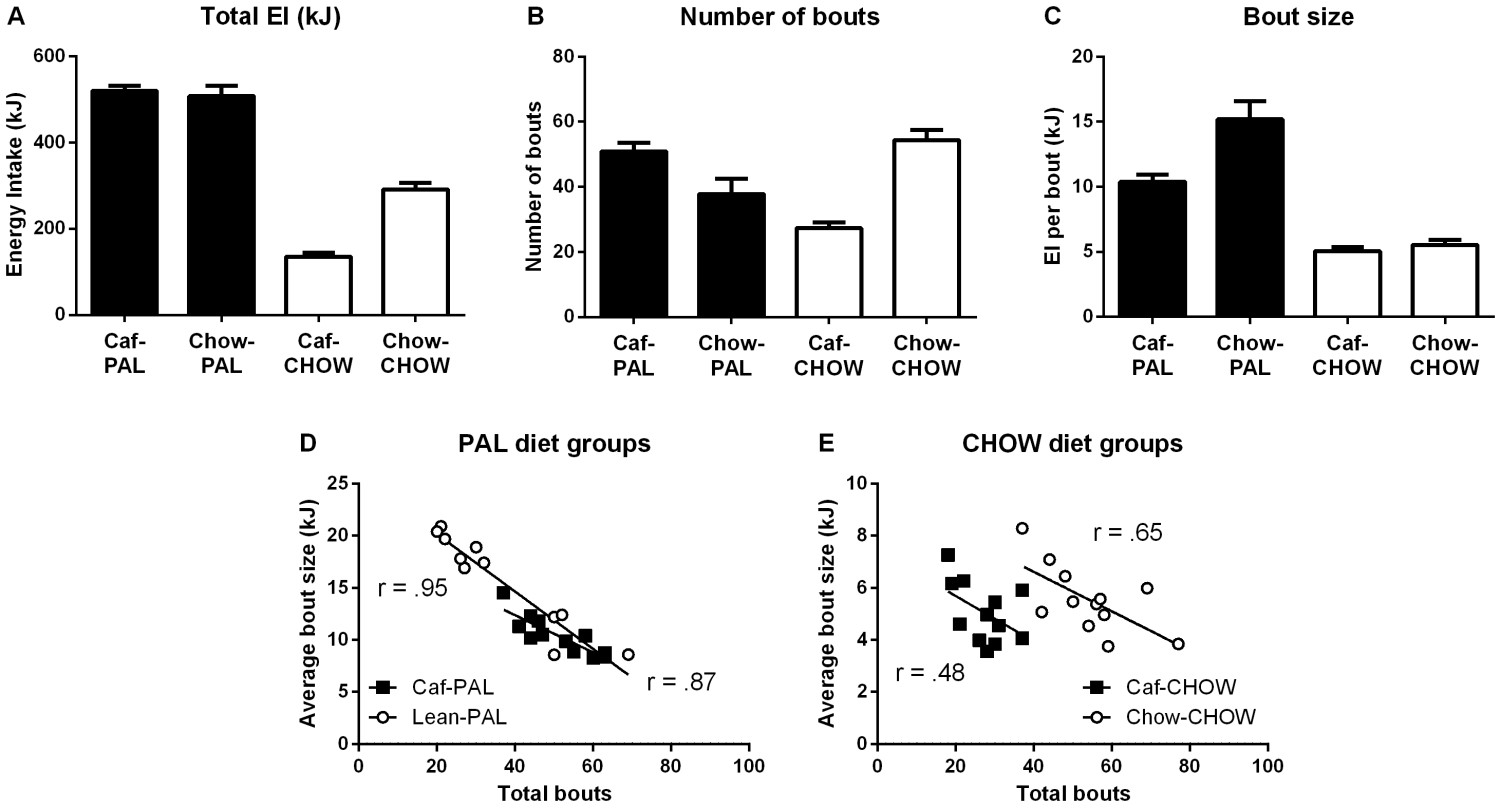
Cross-week comparison of switch effects. (**A–C**) Total energy intake (kJ, A), numbers of bouts (B) and bout size (kJ per bout, C) for Caf-PAL rats in week 1 versus Chow-PAL rats in week 7 (left half of each figure), and Chow-CHOW rats in week 1 versus Caf-CHOW rats in week 7 (right half of each figure). Each column represents the mean (+SEM) for an entire 24-hour session. (**D & E**) Total number of bouts versus average bout size (kJ) for individual rats in Groups Caf-PAL (week 1) and Chow-PAL (week 7; 8D), and for individual rats in Groups Caf-CHOW (week 7) and Chow-CHOW(week 1, 8E).

The top panel of Figure 8 shows total energy intake (8A), numbers of bouts (8B) and bout size (energy intake per bout, 8C) for Caf-PAL rats in week 1 versus Chow-PAL rats in week 7 (left half of each figure), and Chow-CHOW rats in week 1 versus Caf-CHOW rats in week 7 (right half of each figure). Caf-PAL and Chow-PAL rats consumed the same amount of energy, *F*<1, but differed in the way this energy was obtained: Chow-PAL rats initiated fewer bouts, *F*(1,22) = 5.88, p<.05, but consumed more energy in each bout, *F*(1,22) = 10.63, p<.01, than Caf-PAL rats. Caf-CHOW rats consumed less energy than Chow-CHOW rats, *F*(1,22) = 75.89, p<.01, which was due to the fact that they initiated fewer bouts, *F*(1,22) = 52.46 p<.01, rather than consuming smaller bouts, *F*<1.

The bottom panel of Figure 8 shows the total numbers of bouts plotted against average bout size for individual rats in Groups Caf-PAL and Chow-PAL (8D) and Groups Caf-CHOW and Chow-CHOW (8E). With the exception of those in Group Caf-CHOW, rats in each of the other three groups showed a clear negative relationship between the number of total bouts and the average size of a bout, smallest *t*(10) = 2.71, p<.025; rats that consumed more energy per bout initiated fewer bouts overall. This relationship was absent among Caf-CHOW rats, *t* = 1.73: the initiation of a bout among these rats was largely independent of how much they in fact consumed during each bout.

## Discussion

This study examined changes in feeding patterns among rats with a history of exposure to a cafeteria-style diet, Caf rats, or standard lab chow, Chow rats. We assessed feeding patterns among these rats using the CLAMS, where rats were presented with foods consistent with their home cage history: specifically, Caf rats were presented with palatable biscuit and high-fat chow while Chow rats were presented with standard lab chow. The unit of analysis was individual eating bouts that occurred during 24-hour test sessions at different stages of diet exposure. This unit of analysis avoided arbitrary classification schemes that, in the absence of independent verification criteria (e.g., such as the behavioural satiety sequence [7]), has the potential to obscure rather than clarify how feeding patterns change with exposure to a cafeteria diet.

Among Chow rats consuming chow, the number of feeding bouts declined over time, the average bout size increased, and there was a clear positive relationship between these measures: those rats that had the largest bout sizes waited the longest until the next bout, suggesting that feeding in these rats was regulated by time since the last bout. Compared to Chow rats, Caf rats consuming palatable biscuit and high fat chow initiated fewer bouts [7], but this difference was transient: by the final test session, Caf and Chow rats initiated feeding bouts with the same regularity. In contrast, Caf rats consumed more energy per bout, and this difference persisted across all sessions of testing. These differences in Caf rats relative to Chow rats were reflected in differences in the distributions of inter-bout intervals and bout sizes: in the first and last test sessions, Caf rats showed a leftward shift in the distribution of inter-bout intervals (they were more likely than Chow rats to initiate feeding in the minutes following a prior bout), and a rightward shift in the distribution of bout sizes (they were less likely than Chow rats to have bouts of size 10 kJ or less). In spite of these shifts, the relationship between average bout sizes and average wait times between bouts among Caf rats was the same as that observed among Chow rats: feeding in both groups of Caf rats was regulated by time since the last bout.

Critically, a history of exposure to the cafeteria-style diet dramatically affected motivation to consume diets of varying palatability or variety. First, Caf rats maintained on the cafeteria diet and provided with standard lab chow in the CLAMS dramatically reduced their consumption. Relative to Chow rats fed standard lab chow throughout the study and subjected to an equivalent test history, this reduced consumption was due to fewer bouts, reflecting a rightward shift in the distribution of waiting times between bouts; as well as a decrease in energy consumed per bout, reflecting a leftward shift in the distribution of bout sizes. Relative to Chow rats fed standard lab chow in the first week of CLAMS testing, the reduced consumption in Caf rats provided with chow was specifically due to fewer bouts: Chow rats tested with chow for the first time and Caf rats tested with chow for the first time consumed bouts of the same size. The change in feeding when Caf rats were withdrawn from the cafeteria diet for the first time was perhaps most evident in the absence of any relationship between average bout size and average wait time between bouts. Hence, feeding in this group was not regulated by the time since the previous bout. One obvious possibility is that the history of exposure to the cafeteria diet rendered standard lab chow relatively unpalatable [3], [11], and so, in spite of long intervals between bouts that likely engendered hunger, these rats refrained from feeding.

Second, as Caf rats accumulated experience with cafeteria-style foods in their home cage, their motivation to consume biscuit and high-fat chow in the CLAMS test sessions declined: these rats ultimately consumed less of these foods compared to Chow rats exposed to them for the first time (in fact, Caf rats presented with biscuit and high fat chow ultimately consumed these foods to the same modest level as Chow rats fed standard lab chow). Comparisons of Caf and Chow rats exposed to the palatable foods revealed that Caf rats fed less regularly, but when they did feed, they consumed the same amount in energy. Consistent with this, the distribution of waiting times between bouts was shifted to the right for Caf rats, but distributions of bout sizes were not different. When consumption of biscuit and high-fat chow was analyzed separately, Chow rats exposed to biscuit and high-fat chow showed no preference for one over the other, consuming both just as avidly; whereas Caf rats preferred biscuit over high-fat chow. It should also be noted that Chow rats showed a clear positive relationship between the average size of high-fat chow bouts and wait times following these bouts; but there was no relationship between the average size of biscuit bouts and wait times following these bouts. In contrast, Caf rats showed a clear positive relationship between the size of high-fat chow bouts and post-high-fat chow wait times, as well as the size of biscuit bouts and post-biscuit wait times. Taken together, these findings have three important implications. First, the fact that feeding in Caf but not Chow rats following a biscuit bout was regulated by time since the last biscuit bout suggests at least some degree of adaptation to the cafeteria diet. Second, possibly as a consequence of this adaptation, the motivation to consume palatable foods declined among rats exposed to the cafeteria diet. Third, exposure to the cafeteria diet shifted food preferences.

Why did motivation to consume biscuit and high-fat chow decline among Caf rats? There are a number of possible explanations. First, studies have shown that variety in the diet enhances meal size and energy intake among rats exposed to palatable foods [4]. Having been exposed to a range of foods that differed in flavor and texture in their home cages, Caf rats were accustomed to variety, and hence, the relative lack of variety when rats were tested in the CLAMS may have been enough to reduce motivation to feed. Second, rats given chronic access to a cafeteria-style diet become relatively insensitive to reward, indexed by an increase in the threshold at which direct electrical self-stimulation of neural reward circuits was maintained relative to control rats exposed to standard lab chow [2]. The implication of these blunted neural responses is that palatable foods that are normally highly valued and rewarding lose their ability to initiate and maintain behaviors directed towards their procurement.

Diet-induced changes in the rewarding value of palatable foods may occur either because of, or in addition to, increases in circulating levels of anorectic hormones, and therefore, the satiating capacity of energy dense foods. In this respect, previous work in our lab has shown that following two weeks of exposure to the same cafeteria diet as that used in the current experiment, levels of blood glucose and inhibitory feeding signals, insulin and leptin, are significantly elevated [5]. The effects of blood glucose changes on insulin release and its subsequent anorectic effects are well established [12]. In humans, obesity is associated with reduced levels of the stimulatory feeding signal ghrelin [13], while dieting is associated with increases in ghrelin [14]. Ghrelin levels are differentially affected by consumption of different macronutrient components: release of ghrelin is enhanced following consumption of meals containing fat and protein, but reduced by consumption of carbohydrate [15]. Hence, high fat meals have poorer satiation potential compared to high carbohydrate meals [16]–[18], resulting in less frequent but larger meals [19]–[20].

The fact that rats in the current study were exposed to just two foods during CLAMS test sessions makes it difficult to make claims about seeking of particular macronutrients (e.g., protein). Nonetheless, the fact that Caf, but not Chow rats demonstrated a preference for biscuit over high-fat chow suggests that chronic exposure to the cafeteria diet biases selection of foods that are higher in fat, carbohydrate, and overall energy (biscuit). This suggestion is consistent with a wealth of evidence showing that obese people prefer palatable foods, such as those rich in sugar [21]. It is also consistent with evidence for meal-skipping and increased snacking on energy-rich foods (those with high and fat content) among adolescents and young adults [22]–[23]. Thus, the increased prevalence of obesity may be accompanied by an increased prevalence of irregular meal patterns.

While diet-induced changes in food palatability explain many aspects of the present findings, other factors may have also played a role. According to the “Protein Leverage Hypothesis”, energy intake is tightly regulated by the levels of protein in available foods, rather than levels of carbohydrate or fat [24]–[26]. Consistent with this hypothesis, Chow rats accustomed to eating standard lab chow in the home cage and exposed to foods relatively low in protein in the CLAMS (e.g., biscuit and high-fat chow) may have consumed more of the test foods to obtain their usual intake of protein. However, it does not explain why Caf rats accustomed to eating palatable foods in the home cage and exposed to a relatively rich source of protein in the CLAMS (i.e., standard lab chow) consumed less than their Chow counterparts: if the Caf-CHOW rats were simply protein seeking, one may have expected that their energy intake in the CLAMS test sessions should have been equivalent to that of the Chow-CHOW rats.

In summary, the present study has extended previous studies showing that exposure to a cafeteria diet alters feeding patterns such that bout numbers decrease [7], while the energy density of each bout increases. It has also shown that exposure to the diet increases preferences for food that is relatively rich in fat and carbohydrate (biscuit over high-fat chow); and dramatically reduces motivation to consume palatable foods, at least under circumstances where variety is limited. Finally, it has shown that complete withdrawal from cafeteria-style foods diminishes the motivation to feed, and that what feeding does occur is not regulated by factors like time since the last bout [10]. Extrapolation of these findings to people suggests that the affordability, attractiveness and availability of palatable energy dense foods may initially encourage consumption of larger snacks and meals, in terms of both volume, and critically, total energy content. However, over time, it may be the extraordinary variety of energy dense foods that drives over-consumption, and therefore, excessive weight gain.
